# Manual of AnteNatal Pathology and Hygiene—The Fœtus

**Published:** 1903-03

**Authors:** 


**Manual of AnteNatal Pathology and Hygiene—The Fœtus.** By J. W. Ballantyne, M.D. Pp. xvi., 527. Edinburgh: William Green & Sons. 1902.

The field of ante-natal pathology is probably almost a *terra incognita* to most medical men. A book such as this, however, shows how large is the literature upon this subject, and how much still remains to be known. The subject of the infection of the fœtus through the mother is an interesting one; and some of us may be unaware that small-pox, measles, and typhoid fever have all been transmitted from the mother to the unborn infant. There are some interesting points also in connection with vaccination: thus, of fourty-four women who were vaccinated without success during their pregnancies, presumably on account of earlier successful vaccination, thirty-one gave birth to infants that were refractory to vaccination.

One question that affects Bristol is the influence of tobacco upon the fœtus. Some writers appear to think that not only are women-workers in tobacco factories more likely to abort than other women, but "that there is a very large infantile mortality in post-natal life among the offspring of women-workers in tobacco." Manufactures in which salts of lead are employed also affect the life of the fœtus. The well-known deleterious effects of over-indulgence in alcohol in the parents are also discussed.

Besides such familiar conditions as the various aspects of congenital syphilis, one will find the chief facts that are known about such diseases as fœtal dropsy, elephantiasis, fœtal bone disease, fœtal ascites, abnormal conditions of the bladder, ureters, and kidneys. The causation of hydramnios is also discussed, as well as the pathology of fœtal death. Much though the book contains, one is surprised to find that even here all abnormal congenital disease conditions do not appear to be referred to; yet there is little doubt that should anyone wish to find information about some abnormality of the fœtus with which he has become acquainted, he is not likely to consult this book in vain.

